# Alterations to the bovine bacterial ocular surface microbiome in the context of infectious bovine keratoconjunctivitis

**DOI:** 10.1186/s42523-023-00282-4

**Published:** 2023-11-23

**Authors:** Hannah B. Gafen, Chin-Chi Liu, Nikole E. Ineck, Clare M. Scully, Melanie A. Mironovich, Christopher M. Taylor, Meng Luo, Marina L. Leis, Erin M. Scott, Renee T. Carter, David M. Hernke, Narayan C. Paul, Andrew C. Lewin

**Affiliations:** 1 Department of Veterinary Clinical Sciences, Louisiana State University, Skip Bertman Drive, Baton Rouge, LA 70803 USA; 2 Department of Microbiology, Immunology, and Parasitology, School of Medicine, Louisiana State University, 2020 Gravier St, New Orleans, LA 70112 USA; 3 Department of Small Animal Clinical Sciences, Western College of Veterinary Medicine, 52 Campus Dr, Saskatoon, SK S7N 5B4 Canada; 4 Department of Clinical Sciences, College of Veterinary Medicine, Cornell University, 602 Tower Rd, Ithaca, NY 14853 USA; 5 Department of Ambulatory Medicine and Theriogenology, Cummings School of Veterinary Medicine, Tufts University, 200 Westboro Rd, North Grafton, MA 01536 USA; 6 Texas A&M Veterinary Medical Diagnostic Laboratory, Texas A&M University, 483 Agronomy Rd, College Station, TX 77843 USA

**Keywords:** Bovine, Moraxella bovis, 16S rRNA gene sequencing, Real-time PCR, Bacterial culture, Microbiome, IBK, Infectious bovine keratoconjunctivitis, Pink eye, Cattle

## Abstract

**Background:**

Infectious bovine keratoconjunctivitis (IBK) is a common cause of morbidity in cattle, resulting in significant economic losses. This study aimed to characterize the bovine bacterial ocular surface microbiome (OSM) through conjunctival swab samples from Normal eyes and eyes with naturally acquired, active IBK across populations of cattle using a three-part approach, including bacterial culture, relative abundance (RA, 16 S rRNA gene sequencing), and semi-quantitative random forest modeling (real-time polymerase chain reaction (RT-PCR)).

**Results:**

Conjunctival swab samples were obtained from eyes individually classified as Normal (n = 376) or IBK (n = 228) based on clinical signs. Cattle unaffected by IBK and the unaffected eye in cattle with contralateral IBK were used to obtain Normal eye samples. *Moraxella bovis* was cultured from similar proportions of IBK (7/228, 3.07%) and Normal eyes (1/159, 0.63%) (*p* = 0.1481). *Moraxella bovoculi* was cultured more frequently (*p* < 0.0001) in IBK (59/228, 25.88%) than Normal (7/159, 4.40%) eyes. RA (via 16 S rRNA gene sequencing) of *Actinobacteriota* was significantly higher in Normal eyes (*p* = 0.0045). *Corynebacterium variabile* and *Corynebacterium stationis* (*Actinobacteriota*) were detected at significantly higher RA (*p* = 0.0008, *p* = 0.0025 respectively) in Normal eyes. *Rothia nasimurium* (*Actinobacteriota*) was detected at significantly higher RA in IBK eyes (*p* < 0.0001). Alpha-diversity index was not significantly different between IBK and Normal eyes (*p* > 0.05). Alpha-diversity indices for geographic location (*p* < 0.001), age (*p* < 0.0001), sex (*p* < 0.05) and breed (*p* < 0.01) and beta-diversity indices for geographic location (*p* < 0.001), disease status (*p* < 0.01), age (*p* < 0.001), sex (*p* < 0.001) and breed (*p* < 0.001) were significantly different between groups. Modeling of RT-PCR values reliably categorized the microbiome of IBK and Normal eyes; primers for *Moraxella bovoculi*, *Moraxella bovis*, and *Staphylococcus spp.* were consistently the most significant canonical variables in these models.

**Conclusions:**

The results provide further evidence that multiple elements of the bovine bacterial OSM are altered in the context of IBK, indicating the involvement of a variety of bacteria in addition to *Moraxella bovis*, including *Moraxella bovoculi* and *R. nasimurium*, among others. *Actinobacteriota* RA is altered in IBK, providing possible opportunities for novel therapeutic interventions. While RT-PCR modeling provided limited further support for the involvement of *Moraxella bovis* in IBK, this was not overtly reflected in culture or RA results. Results also highlight the influence of geographic location and breed type (dairy or beef) on the bovine bacterial OSM. RT-PCR modeling reliably categorized samples as IBK or Normal.

**Supplementary Information:**

The online version contains supplementary material available at 10.1186/s42523-023-00282-4.

## Background

Infectious bovine keratoconjunctivitis (IBK), colloquially termed “pinkeye”, is the most common ophthalmic disease of cattle [1] and a major cause for morbidity in this species [2]. First described in the late 1800s [3, 4], this condition continues to be identified in the United States and globally [5]. IBK manifests clinically as blepharospasm, epiphora, corneal edema, vascularization, and corneal ulceration, and may progress to corneal perforation and subsequent vision loss [2, 6]. Pain and vision loss associated with this condition ultimately lead to decreased weight gain [1] and decreased milk production [6]. IBK is not only an animal welfare concern but also leads to economic losses for producers at slaughter [7, 8]. Major economic losses, most recently reported at 150 million USD annually [9], are incurred by producers as a result of treatment costs and loss of value due to decreased weight gain, decreased milk production, and corneal scarring [2].

Though more recently suggested to be an umbrella-term for a range of seemingly indistinguishable ocular diseases in cattle [2, 5], IBK is historically thought to be caused by *Moraxella bovis*, a gram-negative coccobacillus bacteria [6, 10]. This organism is the only organism to consistently produce IBK-like lesions in various experimental models when combined with corneal scarification [11–13]. IBK infection has also been associated with the presence of *Moraxella bovoculi* [14], *Mycoplasma spp.* [15], and other pathogenic and opportunistic pathogens, though a definitive causal relationship has not been established [16]. Treatment therefore often includes parenteral broad-spectrum antibiotics labeled for use in cattle with IBK [1, 6]. Vaccinations specific to these organisms as preventative measures have been produced and administered with little to no experimentally proven efficacy [6, 17, 18].

The bacterial ocular surface microbiome (OSM), though low in biomass, has been studied both in the eyes of normal animals [19–24] and in the context of disease in humans [25–27]. Recognizing differences in the composition of the OSM in normal and diseased states allows identification of unique targets which may be exploited for treatment and prevention of disease.

Various methods of assessing the OSM are available including bacterial culture, 16 S ribosomal ribonucleic acid (rRNA) gene sequencing, and real-time polymerase chain reaction (RT-PCR). Bacterial culture using ocular surface samples has been shown to have relatively low diagnostic utility, particularly when compared to results achieved by molecular diagnostic tools [28]. For example, suspected false-negative bacterial cultures have been identified in IBK cases, and therefore alternative or adjunctive testing is recommended [10]. 16 S rRNA gene sequencing utilizes conserved and hypervariable regions of bacterial genetic material to detect and classify a broad range of bacteria into amplicon sequence variants (ASVs), which are then used to evaluate relative taxonomic diversity richness and evenness of distribution through alpha- and beta-diversity indices [29–32]. However, assessing relative composition by use of sequencing alone may lead to incomplete characterization of the impact of mutual dependence and thus interaction of taxa [33, 34]. In addition, 16 S rRNA gene sequencing is associated with higher costs and requires performance of advanced bioinformatic techniques [35]. Real-time polymerase chain reaction (RT-PCR) for semi-quantitative assessment of specific elements of bacterial ocular microbiome through amplification of primer-specific signals [32, 36], on the other hand, is recognized for its relative simplicity, accessibility, and cost-effectiveness [33]. Clinically, quantitative PCR has been employed to create a mathematical model and subsequently a single numerical value, known as the dysbiosis index, to provide actionable information regarding changes to a patient’s microbiome [35, 37, 38]. However, drawbacks to RT-PCR when compared to 16 S rRNA gene sequencing include a limited scope due to the selection of specific bacterial primers for assessment, as well as increased labor and time [39]. Yet relative abundance through sequencing and quantitative assessment through PCR may be considered complementary when studying the microbiome; sequencing provides a broad overview and suggests possible PCR primer targets, while quantitative PCR provides repeatable, quantitative data and allows identification of biases in analysis [39]. Combining the techniques of bacterial culture, 16 S rRNA gene sequencing, and semi-quantitative RT-PCR when evaluating a specific microbiome may provide both a thorough overview and in-depth picture, allowing for a deeper understanding of the community under study. More specifically, wholly understanding microbiome dynamics may yield identification of the most appropriate target organism for treatments in the context of dysbiosis [33].

Reports investigating the bovine bacterial OSM through bacterial culture, 16 S rRNA sequencing or PCR in the context of IBK exist. While historically *Moraxella bovis* was isolated most commonly in cases of IBK [7], one retrospective study found that a majority of IBK samples (600/1042) yielded viable *Moraxella bovoculi* [40]. Another study of calves naturally infected with IBK found through PCR testing that *Moraxella bovoculi* was more commonly detected than *Moraxella bovis* [41]. A separate study utilized 16 S rRNA gene sequencing of calves naturally infected with IBK and reported minimal detectable differences in microorganism abundance between affected and control animals [42]. Finally, a longitudinal study of 16 S rRNA gene sequencing of calves naturally infected with IBK identified increased relative abundance of *Mycoplasma* and decreased relative abundance of *Moraxella* in the context of IBK [9]. To the authors’ knowledge, a study combining the three techniques of culture, 16 S rRNA gene sequencing, and RT-PCR has yet to be performed and may more completely elucidate microbiome dynamics in the context of IBK.

The purpose of this study was to comprehensively characterize the bovine bacterial OSM in the naturally-occurring IBK disease state as compared to Normal through a combination of bacterial culture, 16 S rRNA gene sequencing, and RT-PCR. We hypothesized that each of the three utilized methods would detect significant differences in the bovine bacterial OSM based on disease status (IBK or Normal).

## Results

### Demographic data

Six hundred and four individual eyes (Table 1) from cattle from 8 states (Table 2) and 17 farms (Table 3) were included in the study. Two hundred and twenty-eight eyes were diagnosed as having IBK disease, while 376/604 eyes were diagnosed as Normal. Chi-squared test was used to assess for significance of demographic data. There was no correlation identified between eye sampled (OS or OD) and disease status (Normal or IBK) (*p* = 0.1652). A higher percentage of IBK eyes compared to Normal eyes were sampled from male cattle (54/109, 49.54% IBK) than were sampled from female cattle (174/495 eyes, 35.15% IBK) (*p* = 0.0055). Age (three categories: < 1 year old, 1 to 5 years old, and > 6 years old) was independent of disease status (Normal or IBK) (*p* = 0.1885). Most eye sampled (573/604) originated from animals aged < 1 year old, which reflects the nature of meat and dairy production in the USA. Breed, when classified into one of two categories (those raised for beef or dairy purposes), was independent of disease status (*p* = 0.9353).

**Table 1:** Demographic data, including total samples collected, and variables recorded such as eye, sex, age, breed purpose, and individual breeds. Chi-squared test was used to assess for significance

Category	Total Number of Eyes	Number of IBK Eyes	Number of Normal Eyes	Statistical Significance (Chi-Squared)
**Total Number of Eyes**	604	228(37.75%)	376(62.25%)	
**Eye Sampled**				
Right eye	296	120(40.54%)	176(59.46%)	*P* = 0.1652
Left eye	308	108(35.06%)	200(64.94%)	
**Sex**				
Female	495	174(35.15%)	321(64.85%)	*P* = 0.0055
Male	109	54(49.54%)	55(50.46%)	
**Age**				
< 1 year old	573	221(38.57%)	352(61.43%)	*P* = 0.1885
1–5 years old	24	5(20.83%)	19(79.17%)	
> 6 years old	7	2(28.57%)	5(71.43%)	
**Purpose/Breed**				
**Beef cattle**	388	146(37.63%)	242(62.37%)	
Angus	371	140 (37.74%)	231(62.26%)	*P* = 0.9353
Hereford	15	5(33.33%)	10(66.66%)	
Charolais	2	1(50%)	1(50%)	
**Dairy cattle**	216	82(37.96%)	134 (62.04%)	
Brown Swiss	8	4(50%)	4(50%)	*P* = 0.9353
Holstein	202	77(38.11%)	125(61.88%)	
Jersey	6	1(16.67%)	5(83.33%)	

**Table 2:** Location data by US state, including total samples collected

Location (State)	Total Number of Eyes	Number of IBK Eyes	Number of Normal Eyes
Connecticut	103	38	65
Georgia	47	8	39
Idaho	125	69	56
Louisiana	6	3	3
Pennsylvania	20	5	15
Vermont	113	44	69
Virginia	41	7	34
West Virginia	149	54	95

**Table 3:** Location data by farm, including total number of eyes sampled

Location (Farm)	Total Number of Eyes	Number of IBK Eyes	Number of Normal Eyes
Farm 1	26	3	23
Farm 2	21	5	16
Farm 3	27	3	24
Farm 4	65	26	39
Farm 5	24	5	19
Farm 6	125	69	56
Farm 7	78	42	36
Farm 8	19	4	15
Farm 9	14	3	11
Farm 10	41	7	34
Farm 11	11	2	9
Farm 12	4	2	2
Farm 13	2	1	1
Farm 14	31	9	22
Farm 15	38	12	26
Farm 16	58	30	28
Farm 17	20	5	15

### Bacterial culture

In total, 387 culture swabs were obtained from 228 IBK eyes and 159 Normal eyes for in-vitro culture (Table 4). Associations were investigated by Chi-squared test. There were no significant differences in the frequency of positive *Moraxella bovis* isolation rates between groups (IBK or Normal) (*p* = 0.1481). There were significant differences in the frequency of positive *Moraxella bovoculi* isolation rates between groups (IBK or Normal) (*p* < 0.0001). There were no significant differences in the frequency of positive *Moraxella osloensis, Proteus spp., Trueperella pyogenes*, unspecified fungus, *Bacillus spp.*, and mixed bacterial growth cultures between groups (IBK or Normal) (*p* > 0.05).

**Table 4:** Aerobic bacterial culture results. Chi-squared test was used to assess for significance

Category	IBK Eyes: Positive Culture	IBK Eyes: Negative Culture	Normal Eyes: Positive Culture	Normal Eyes: Negative Culture	Statistical significance (Chi-squared)
*Moraxella bovis*	7(3.07%)	221 (96.93%)	1(0.63%)	158(99.37%)	*P* = 0.1481
*Moraxella bovoculi*	59(25.88%)	169(74.12%)	7(4.40%)	152(95.60%)	*P* < 0.0001
*Moraxella osloensis*	1(0.44%)	227(99.56%)	0(0%)	159(100%)	*P* > 0.05
*Trueperella pyogenes*	4(1.75%)	224(98.25%)	0(0%)	159(100%)	*P* > 0.05
Unspecified fungus	10(4.39%)	218(95.61%)	12(7.55%)	147(92.45%)	*P* > 0.05
*Bacillus spp.*	7(3.07%)	221(96.93%)	2(1.26%)	157(98.74%)	*P* > 0.05
*Proteus spp.*	1(044%)	227(99.56%)	0(0%)	159(100%)	*P* > 0.05
Mixed bacterial growth	220(96.49%)	8(4.51%)	156(98.11%)	3(1.89%)	*P* > 0.05

### 16 S rRNA gene sequencing: bacterial population composition

**Fig. 1:**
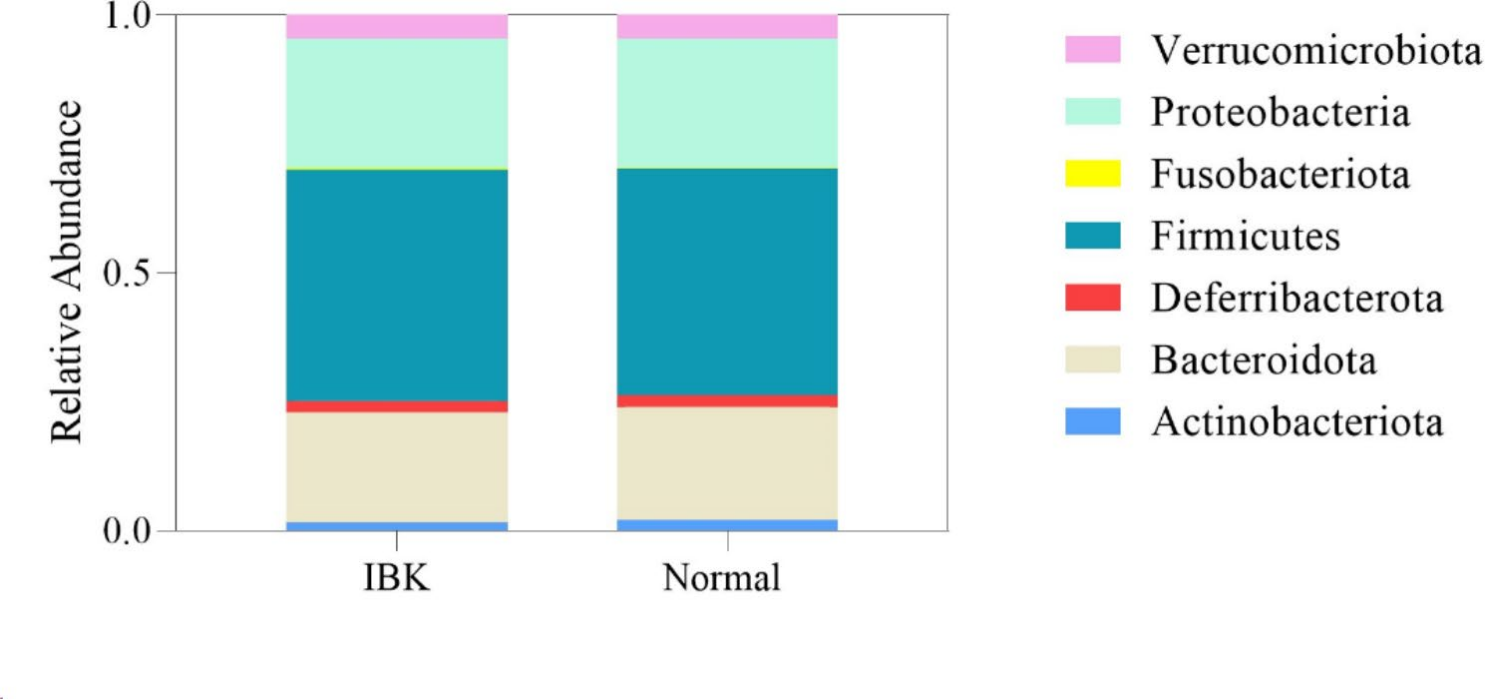
Relative abundance of bacteria at the phylum level based on 16 S rRNA gene sequencing of 228 IBK eyes and 367 Normal eyes (604 samples). The seven most commonly identified phyla encompassing 120 ASVs on the conjunctival surface of IBK and Normal eyes were *Actinobacteriota*, *Bacteroidota*, *Deferribacterota*, *Firmicutes*, *Fusobacteria*, *Proteobacteria*, and *Verrucomicrobiota*

From 604 total conjunctival samples, 21,879 ASVs were determined using DADA2. Decontam was used to remove potential contaminant ASVs and ASVs encompassing less than 0.001% relative abundance were removed, leaving 20,191 ASVs for downstream analysis. The average number of high-quality reads per sample was 22,416 (Supplemental Table 1). The seven most commonly identified phyla from all samples, encompassing 120 ASVs, were*Actinobacteriota, Bacteroidota, Deferribacterota, Firmicutes, Fusobacteriota, Proteobacteria* and *Verrucomicrobiota* (Fig. 1). Student’s t tests or ANOVA tests were used to evaluate relative abundance.

**Fig. 2:**
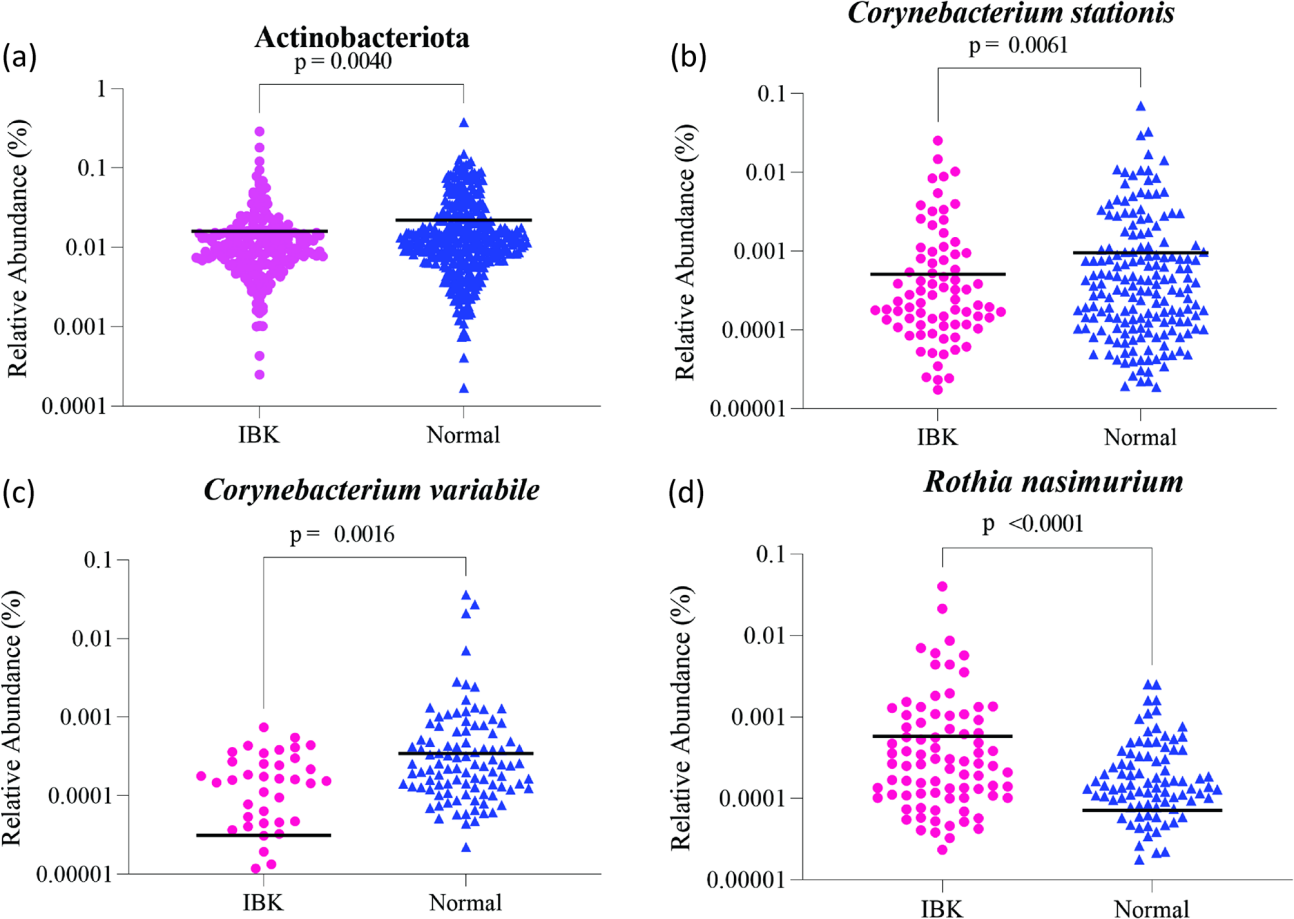
(**a**) The overall relative abundance of *Actinobacteriota* was significantly greater in Normal than IBK eyes (*p* = 0.0040). Three species within *Actinobacteriota* including *Corynebacterium stationis* (*p* = 0.0061) (**b**) and *Corynebacterium variabile* (*p* = 0.0016) (**c**) were noted to present at higher relative abundance in Normal eyes than IBK eyes. A third species within *Actinobacteriota*, *Rothia nasimurium* (**d**), was present at higher relative abundance in IBK eyes (*p* < 0.0001). Student’s t tests or ANOVA tests were used to evaluate abundance

The overall relative abundance of *Actinobacteriota* was significantly greater (*p* = 0.0045) in Normal eyes (mean ± SD: 2.25% ± 3.09%) than IBK eyes (mean ± SD: 1.61% ± 2.61%) (Fig. 2). Species within the *Actinobacteriota* phylum found at significantly higher relative abundance in Normal eyes than in IBK eyes included *Corynebacterium stationis* (mean ± SD: Normal = 0.098% ± 0.46%, IBK = 0.051%± 0.22%; *p* = 0.0025) and *Corynebacterium variabile* (mean ± SD: Normal = 0.034% ± 0.25%, IBK = 0.0031% ± 0.099%; *p* = 0.0008) (Fig. 2). *Rothia nasimurium*, also within the *Actinobacteriota* phylum, was found at significantly higher relative abundance in IBK eyes (mean ± SD: 0.058% ± 0.31%) than in Normal eyes (mean ± SD: 0.0074% ± 0.025%) (*p* < 0.0001) (Fig. 2).

**Fig. 3:**
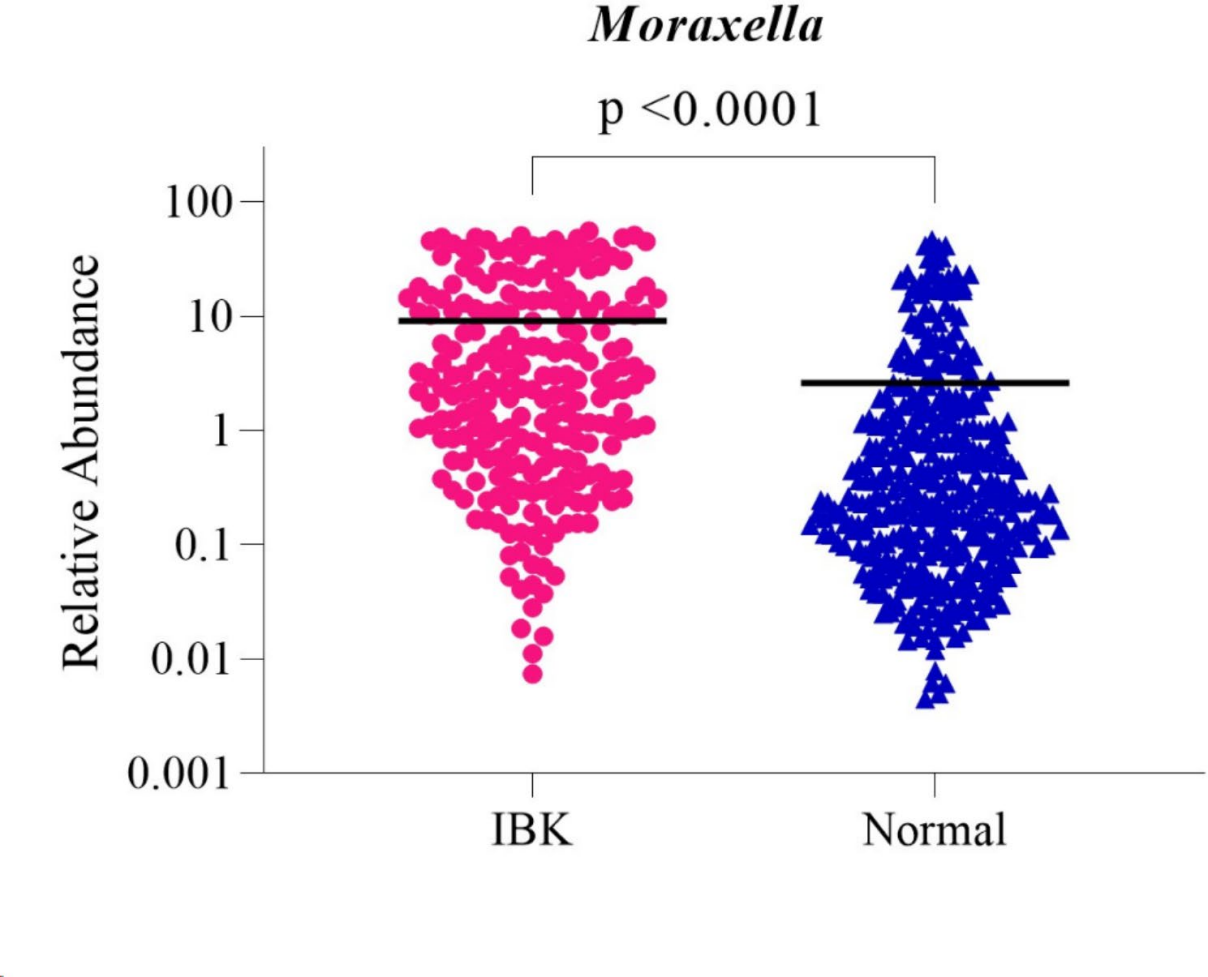
The overall abundance of the *Moraxella* genus was significantly higher in IBK eyes compared with Normal eyes (*p* < 0.0001). Student’s t tests or ANOVA tests were used to evaluate differences in relative abundance

The relative abundance of the *Moraxella* genus (of phylum *Proteobacteria*) was significantly higher in IBK eyes (mean ± SD: 9.15% ± 13.65%) compared with Normal eyes (mean ± SD: 2.62% ± 6.59%) (*p* < 0.0001) (Fig. 3). The relative abundance of the *Pasteurellaceae* (of phylum *Pseudomonadota*) was significantly higher in IBK eyes (mean ± SD: 13.83% ± 1.38%) compared with Normal eyes (mean ± SD: 0.75% ± 0.07%) (*p* = 0.0163).

Significant differences in relative abundance based on geographic location, sex, and breed (by purpose and individual breed) were noted. Relative abundance by geographic location (state and farm, evaluated separately) was significantly different for 7/7 of the most commonly identified phyla (*p* < 0.0001) (Supplemental Tables 2 and 3). Individual breed (6 levels) was also a significant factor for 7/7 of the most commonly identified phyla (*Verrucomicrobiota*: *p* = 0.0007; all other phyla: *p* < 0.0001) (Supplemental Table 4). Relative abundance by breed purpose (dairy or beef) was found to be significantly different for 7/7 phyla (Supplemental Table 5): relative abundance of *Firmicutes* and *Actinobacteriota* were increased in dairy cattle compared to beef cattle (*p* < 0.0001); relative abundance of *Bacteriodota*, *Deferribacterota*, and *Fusobacteriota* were increased in beef cattle (*p* < 0.0001); relative abundance of *Proteobacteria* (*p* = 0.0012) and *Verrucomicrobiota* (*p* = 0.0004) were increased in beef cattle. Relative abundance by sex was noted to be significantly different for 4/7 phyla: *Actinobacteriota* (*p* = 0.0007), *Firmicutes* (*p* < 0.0001), and *Verrucomicrobiota* (*p* = 0.0175) were of higher relative abundance in female cattle (mean ± SD: 2.19% ± 3.08%, 46.0% ± 20.21%, and 4.67% ± 2.57%, respectively) than male cattle (mean ± SD: 1.15% ± 1.89%, 36.4% ± 17.33%, and 4.03% ± 2.49%, respectively), while *Proteobacteria* (*p* < 0.0001) were of higher relative abundance in male cattle (mean ± SD: 35.40% ± 22.45%) than female cattle (mean ± SEM: 22.92% ± 19.10%).

### 16 S rRNA gene sequencing: alpha-diversity

**Fig. 4:**
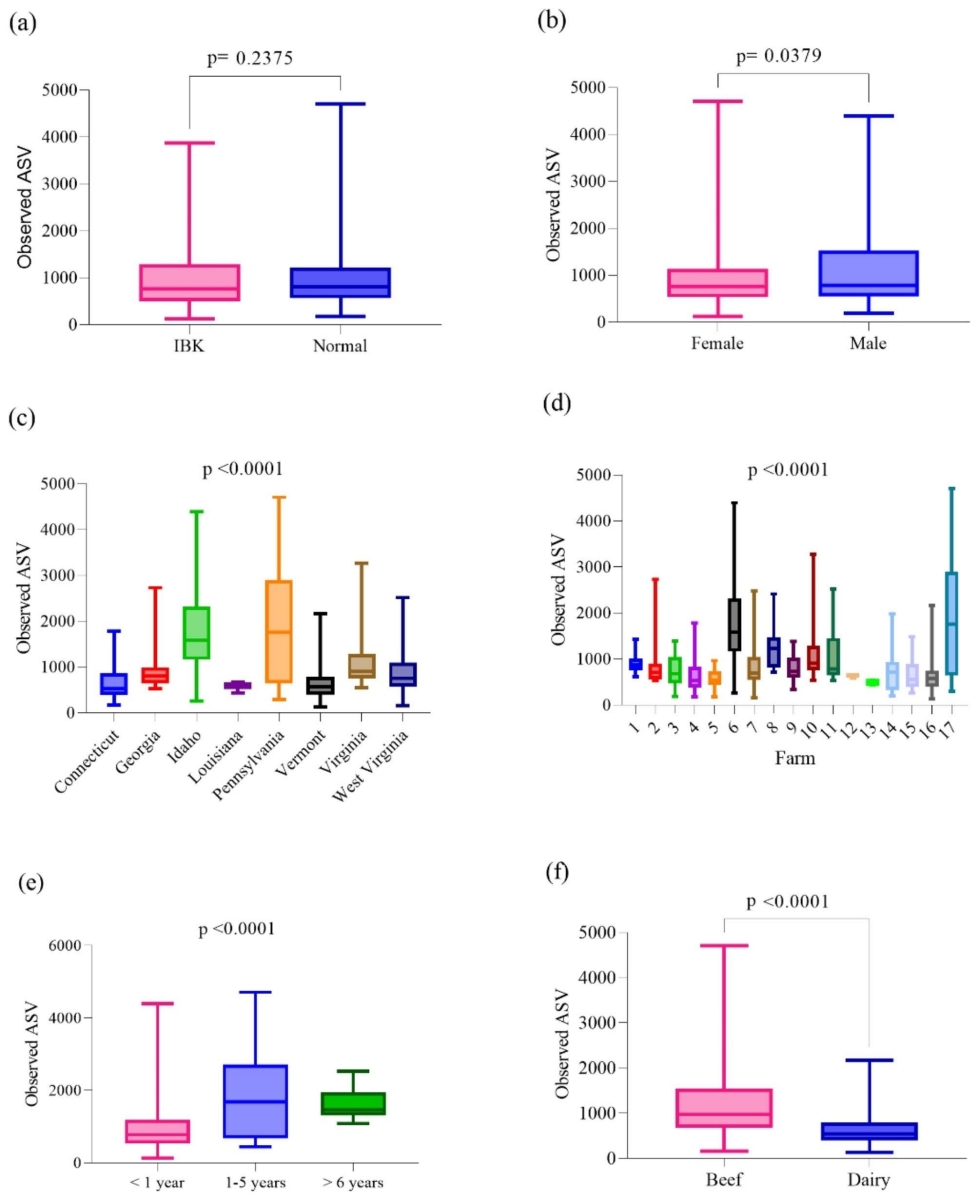
Observed ASV against disease status (**a**), sex (**b**), US state (**c**), farm (**d**), age (**e**), and breed purpose (**f**) evaluated with non-parametric Kruskal-Wallis and Mann-Whitney U tests. No significant differences in alpha-diversity with regard to disease status were observed with Obs ASV (*p* > 0.05). Significant differences in alpha-diversity were detected with regard to geographic location (both by state and farm, *p* < 0.0001), age (*p* < 0.0001), sex (*p* = 0.0379), and breed (*p* < 0.0001) for Obs ASV

Non-parametric Kruskal-Wallis and Mann-Whitney U tests were utilized for the investigation of alpha-diversity (Fig. 4). There were no significant differences in alpha-diversity with regard to disease status (IBK or Normal) via any of the investigated indices, including Observed ASV, Chao1, Shannon, Simpson, and Faith’s PD (*p* > 0.05). There were significant differences in alpha-diversity with regard to geographic location (state and farm) and age by all investigated indices (*p* < 0.0001) and by sex for Observed ASV (*p* = 0.0379), Chao1 (*p* = 0.0456), Pielou (*p* = 0.0207), and Faith’s PD (*p* = 0.0187). There were significant differences in alpha-diversity with regard to breed (dairy and beef) for Observed ASV (*p* < 0.0001), Chao1 (*p* < 0.0001), Shannon (*p* = 0.0003), Simpson (*p* = 0.0181), and Faith’s PD (*p* < 0.0001).

### 16 S rRNA gene sequencing: beta-diversity

**Fig. 5:**
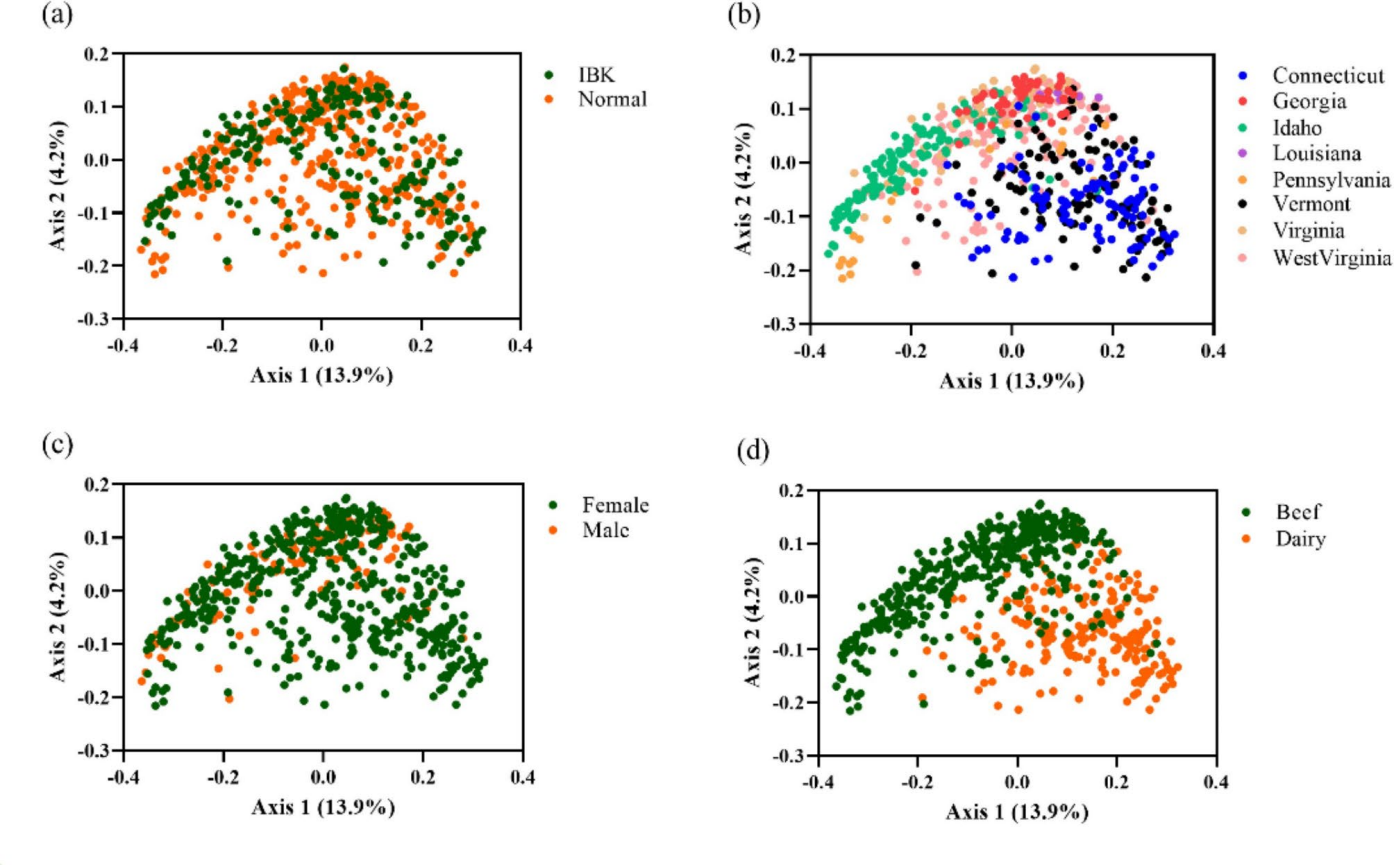
Unweighted unifrac principal coordinate analysis performed by PERMANOVA of disease status (IBK or Normal) (**a**), geographic location (state) (**b**), sex (**c**), and breed (beef and dairy) (**d**). There were significant differences in beta-diversity between IBK and Normal eyes (*p* = 0.002), between geographic locations (state) (*p* = 0.001), between male and female cattle (*p* = 0.001), and between beef and dairy cattle (*p* < 0.001). IBK = infectious bovine keratoconjunctivitis eyes, Normal = normal eyes

There were significant differences in bacterial beta-diversity identified by PERMANOVA by disease status (IBK or Normal) (unweighted unifrac: *p* = 0.002; weighted unifrac analysis: *p* < 0.001; Bray-Curtis: *p* < 0.001; Fig. 5). However, significant differences in beta-diversity were also identified with regard to geographic location (state; unweighted unifrac, weighted unifrac, Bray-Curtis: *p* < 0.001; Fig. 5), sex (unweighted unifrac, weighted unifrac, Bray-Curtis: *p* < 0.001; Fig. 5), age (unweighted unifrac, weighted unifrac, Bray-Curtis: *p* < 0.001; Fig. 5), and breed (beef and dairy: unweighted unifrac, weighted unifrac, Bray-Curtis: *p* < 0.001; Fig. 5). When samples were selectively analyzed in smaller subgroups where the only known variable was disease status, beta diversity indices indicated significantly different microbiome composition in the majority of cases (Supplemental Table 6).

### RT-PCR

Two hundred and twenty-seven IBK and 369 Normal samples were evaluated by RT-PCR. Mann-Whitney U tests were used to evaluate the log DNA concentration against disease status. The average log concentrations of *Moraxella bovis* (IBK/Normal: 5.09/3.73; *p* < 0.0001), *Moraxella bovoculi* (IBK/Normal: 5.14/3.79; *p* < 0.0001), *Pasteurellacaea* (IBK/Normal: 5.37/4.76; *p* < 0.0001), and *Weeksellaceae* (IBK/Normal: 6.38/6.14; *p* = 0.0001) detected were significantly higher in IBK than in Normal eyes (Table 5). The average log concentration of *Staphylococcus spp.* was higher in Normal eyes than in IBK eyes (IBK/Normal: 3.83/4.60; *p* < 0.0001). No significant difference in log concentrations by disease status (IBK or Normal) was observed for the remaining primer targets evaluated (Bov GAPDH, *Mycoplasma*, *Prevotellaceae*, universal bacteria) by RT-PCR (*p* > 0.05) (Table 5).

**Table 5:** Relative DNA quantities obtained by PCR and evaluated by Mann-Whitney U tests. Asterisk (*) indicates significant difference with respect to disease status

	Median (Min-Max) Log DNA (ag = 10^− 18^ g) per 10 ng isolated total DNA	
Target Primer	Disease status	
	Normal (n = 369)	IBK (n = 227)
Bov. GAPDH	7.18 (4.99–8.41)	7.23 (4.85–7.83)
*Moraxella bovis*	3.73 (0.00-6.63)*	5.09 (0.00-8.34)*
*Moraxella bovoculi*	3.79 (0.00-7.10)*	5.14(0.00-7.63)*
*Mycoplasma*	7.20 (3.72–8.85)	7.21(3.74–9.16)
*Staphylococcus*	4.60 (0.00-9.19)*	3.83 (0.00-9.68)*
*Pasteurellaceae*	4.76 (0.00-7.85)*	5.37 (0.00-8.42)*
*Prevotellaceae*	6.01 (4.48–8.41)	6.12(4.64–8.10)
*Weeksellaceae*	6.14 (0.00–9.00)*	6.38 (0.00-8.04)*
Universal bacteria	8.19 (6.63–10.30)	8.23 (6.85–10.30)

### Classification analysis via random forest algorithm

**Fig. 6:**
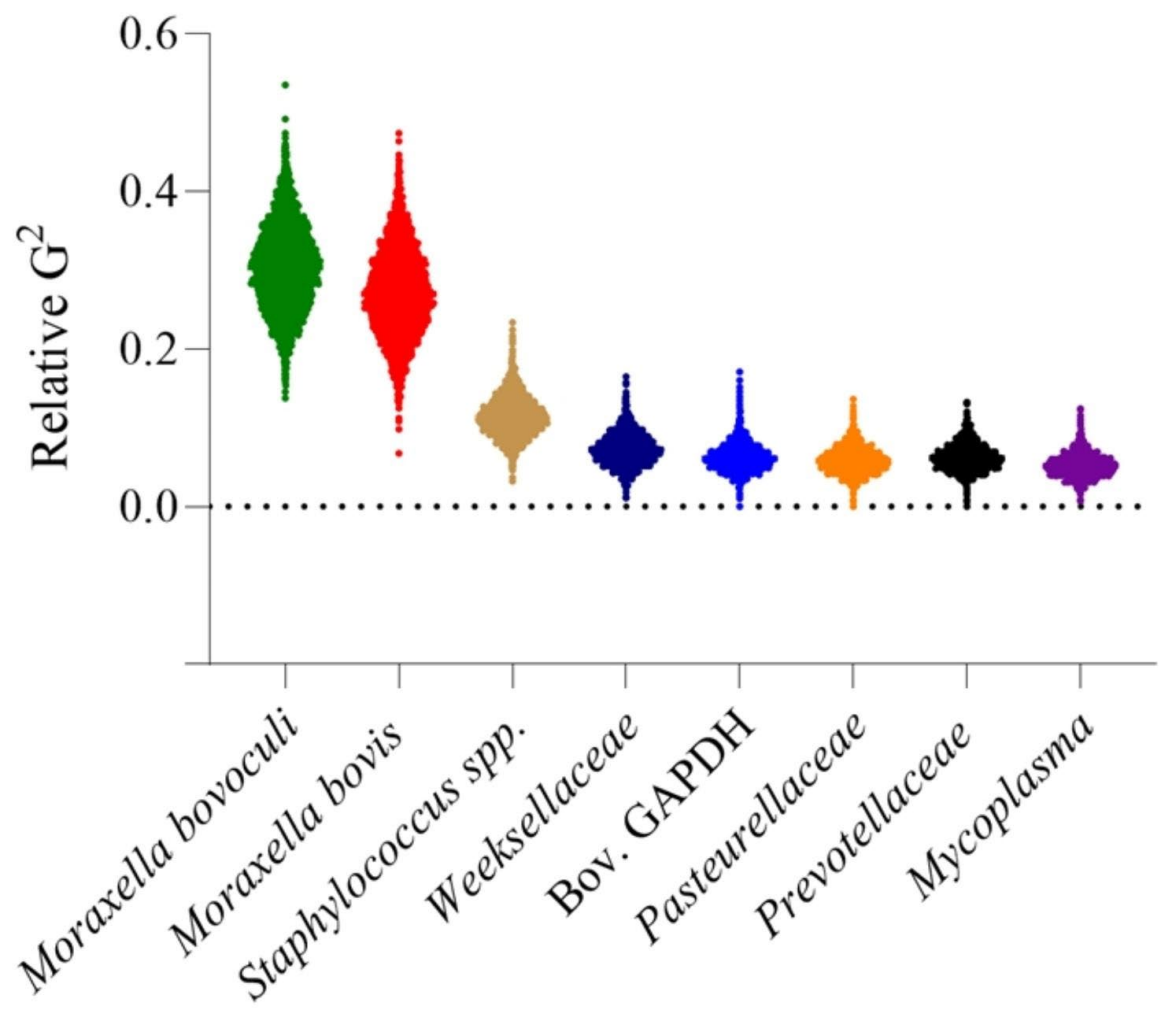
Column contribution of each primer in random forest algorithms presented as relative deviance (G^2^). The three most consistent canonical variables to classify disease status through data modeling of RT-PCR included *Moraxella bovoculi*, *Moraxella bovis*, and *Staphylococcus spp.* The three least significant primers contributing to the canonical variable in the primer set were *Pasteurellaceae*, *Mycoplasma*, and *Prevotellaceae*

Data modeling was performed to categorize RT-PCR values by disease status (IBK or Normal) using training sets, with the resultant model then tested using validation sets. Validation set sensitivities ranged from 69.2 to 95.2%, and specificities ranged from 80.0 to 96.4%. The interpretation of primer contribution was calculated by relative deviance (G^2^) from the individual primer within each trial. The three most consistent primers contributing to the canonical variable to classify disease status were *Moraxella bovoculi* (22.1–42.5%), *Moraxella bovis* (21.2–42.6%), and *Staphylococcus spp.* (9.1–18.5%), while *Pasteurellaceae* (2.8–7.4%) *Mycoplasma* (2.1–6.5%) and *Prevotellaceae* (2.8–7.7%) were consistently the least three significant primers contributing to the canonical variable in the eight-primer set (Fig. 6).

## Discussion

In the present study, a large sample size (n = 604 eyes from 17 farms in 8 US states) was acquired to comprehensively investigate the bovine bacterial OSM through bacterial culture, 16 S rRNA gene sequencing, and RT-PCR to synergistically characterize the OSM in both normal cattle and cattle with IBK. In addition, RT-PCR value modeling was utilized to categorize samples as IBK or Normal. While historically *Moraxella bovis* has been considered the primary etiologic agent associated with IBK, vaccinations and antimicrobial treatments targeting this pathogen show limited efficacy in preventing and limiting disease [6, 17, 18]. The present study confirms alterations in the relative abundance of *Moraxella* species between Normal eyes and IBK eyes but also suggests that numerous additional bovine bacterial OSM changes are present in IBK.

Aerobic bacterial culture was chosen as the first of three methods of OSM assessment due to frequent clinical use for confirmatory diagnosis of IBK [7]. However, results of the present study indicate that bacterial culture alone is unlikely to provide meaningful data for clinical bovine bacterial OSM assessment. Both *Moraxella bovis* and *Moraxella bovoculi* are frequently isolated from both normal and IBK-affected eyes [1]. In the present study, *Moraxella bovis* was isolated using samples from 7/228 (3.07%) of IBK eyes and *Moraxella bovoculi* was isolated using samples from 59/228 (25.88%) of IBK eyes; both organisms were isolated using samples from Normal eyes. As false negatives occur with simultaneous growth of multiple organisms in bacterial culture [10] (220/228 (96.49%) samples from IBK eyes yielded mixed bacterial growth in the present study), this technique has limited diagnostic potential in cattle with IBK. While it is recognized that bacterial culture is an extremely important established technique to identify the presence of viable bacteria, the results of this study indicate that, where possible, additional molecular diagnostic methods should be utilized for the diagnosis of IBK.

16 S rRNA gene sequencing and relative abundance analysis was employed as the second of three methods of bovine bacterial OSM assessment in the context of IBK. Each of the most recently performed studies utilizing 16 S rRNA sequencing to characterize the bovine bacterial OSM concluded that the bovine bacterial OSM is altered in the context of IBK [9, 42, 43]; results of the present study are consistent with this conclusion. The statistically significant proportional differences in bacterial relative abundance between Normal and IBK eyes frequently involved bacterial groups which composed a small proportion of the overall bacterial microbiome. However, it is considered likely that these bacterial groups represent ‘keystone taxa’ which drive community composition and function irrespective of their abundance [44, 45]. Through 16 S rRNA gene sequencing, two species (*Corynebacterium stationis* and *Corynebacterium variabile)* from the phylum *Actinobacteriota* were noted to be present at significantly higher levels in Normal compared to IBK eyes, while a third species under the same phylum (*Rothia nasimurium*) was noted to be present at significantly higher levels in IBK eyes compared to Normal eyes. *Corynebacterium* species are part of the normal flora of the healthy human conjunctival sac and, while considered commensal, certain species have been shown to have pathogenic potential in immunocompromised patients [46]. A study of the murine ocular microbiome reported the protective effect of a *Corynebacterium* species through elicitation of a protective immune response [47]. In cattle, *Corynebacterium stationis* and *Corynebacterium variabil*e have been isolated from the housing and milking environments of normal dairy cows, with *Corynebacterium stationis* most frequently isolated, mainly found in bedding and drinking troughs [48]. Various *Corynebacterium* species have been suggested to have potentially protective probiotic effects in conditions such as nasal dysbiosis [49, 50], vaginal dysbiosis [51, 52], and oral neoplasia [53]. *Corynebacterium* spp. may play a protective role in IBK, and it is suggested that this possibility is explored in future studies. *Rothia nasimurium* is part of the normal flora of the human oropharynx and upper respiratory tract, though *Rothia* species have been implicated as an opportunistic pathogen in serious systemic illnesses in both immunocompromised and immunocompetent patients [54, 55] and have been associated with bacterial endophthalmitis in humans [56, 57]. *Rothia nasimurium* has also been shown to cause systemic disease in chickens [58] and ducks [59]. A study aiming to characterize the bovine ocular microbiota in the context of IBK in Mexico used 16 S rRNA gene sequencing of cultured bacterial isolates and noted the presence of *Corynebacterium* species and *Rothia nasimurium* in some samples among other bacterial species, though the study did not specify if significant differences were detected between normal and IBK eyes [60]. Based on previous findings and the results presented herein, further research to determine the role of *Rothia nasimurium* in IBK is warranted.

The present study found relatively few statistically significant differences in bacterial relative abundance based on disease status (Normal or IBK) using 16 S rRNA gene sequencing. Statistically significant differences in beta-diversity based on disease status were observed, with multiple variables (geographic location, sex, breed, and age) found to influence bovine bacterial OSM diversity. In common with a previous 16 S rRNA gene sequencing ocular microbiome assessment [61], geographic location appeared to significantly influence bovine bacterial OSM diversity in both Normal eyes and IBK eyes in the present study. A possible explanation for this finding is naturally occurring variation between subpopulations and the potential involvement of different IBK etiological agents in different locations. By including eyes from 8 different states and 17 different farms, the present study compiled a large enough sample size and a wide enough sample size to, consequently, describe the influence of geographic location on the bovine bacterial OSM. The context of geographic location must not be overlooked when characterizing the bovine bacterial OSM, and, therefore, all conclusions drawn from the present study must be considered with regard to the location in which samples were collected. In addition, the present study detected significant differences in overall relative abundance and alpha-diversity by sex and breed; therefore, these variables should also be considered when characterizing the bovine bacterial OSM.

A recent study by Anis et al. utilizing 16 S rRNA gene PCR and next generation sequencing analysis found differences in relative abundance of organisms in IBK eyes compared with control eyes [43]. For example, the relative abundance of *Cardiobacteriaceae* and *Pasteurellaceae* were noted to trend higher in eyes with IBK than in control eyes, and control eyes had elevated relative abundance of *Sphingomonadaceae* and *Enterobacterioaceae* compared to IBK eyes [43]. The relative abundance of three of these bacterial families, excluding *Pasteurellaceae*, were not observed to be significantly different between IBK and Normal samples in the present study when assessed using 16 S rRNA gene sequencing. This may be due to differences in 16 S rRNA gene sequencing protocols and analysis, variation of IBK between herds, or relative sample size. It should, however, be noted that the absolute and relative abundance of *Pasteurellaceae* was significantly elevated in IBK eyes compared to Normal eyes when evaluated with 16 S rRNA gene sequencing and RT-PCR, respectively, in the present study. In common with the present study, Anis et al. reported that 16 S rRNA gene sequencing relative abundance assessment of IBK was limited by the inability to distinguish *Moraxella bovis* at the species level [43]. This further highlights the importance of utilizing multiple approaches to characterize the bovine bacterial OSM in the context of IBK.

RT-PCR was utilized as the third and final method of bovine bacterial OSM assessment in the present study. Copy numbers of DNA from *Moraxella bovis*, *Moraxella bovoculi, Pasteurellacea*e and *Weeksellaceae* were significantly higher in IBK eyes compared to Normal eyes. *Staphylococcus spp.* copy numbers were significantly higher in Normal eyes compared to IBK eyes. RT-PCR cycle threshold values were then utilized for classification analysis using a random forest algorithm to categorize eyes by disease status. In addition to possibly representing an improved confirmatory test for IBK, it is possible that this assay could be utilized in the future to identify environmental sources of IBK at individual farms. In the present study, validation set sensitivities and specificities ranged from 69.2 to 95.2%, and 80.0-96.4%, respectively, indicating that samples analyzed with this primer set and mathematical model may predict disease status with moderate to high sensitivity and specificity. A ‘dysbiosis index’ to assess fecal microbiome alterations in veterinary patients, which utilizes a similar approach, has been employed in numerous studies [35, 37, 38, 62]. It is therefore postulated that the present study’s use of classification analysis may be considered for the development of future techniques to study, diagnose, and make treatment recommendations for cattle with IBK.

There are several limitations of the present study, including sample collection techniques, inherent variations in 16 S rRNA gene sequencing and analysis, bacterial culture techniques, validation of assays with no known standard and sample categorization. Samples were collected from cattle naturally affected with IBK while being handled for other management reasons at each farm. Out of necessity, this required rapid sample collection and thorough but brief ocular examinations by a trained veterinarian observer. Gloves were worn and changed between animals, but full, sterile personal protective equipment could not be utilized. We carefully considered this element of study design before performing the study and it was decided that the method of sample collection which was ultimately chosen represented the most realistic approach. As with any study utilizing 16 S rRNA gene sequencing and analysis, variations in sample processing and analysis are likely to have influenced the results. This prevents direct comparison of our results to studies of a similar nature. For this reason, we collected a large sample size from a geographically diverse group of animals. To ensure optimal conditions for transportation of viable bacteria for subsequent in-vitro culture, we worked with experienced bacteriologists to design this element of the study. However, samples did require transportation (chilled) prior to initiation of processing, which could have led to lower numbers of viable bacteria being detected. The Normal samples utilized for bacterial culture originated from animals with contralateral IBK. As such, it is possible that the composition of the bacteria identified using culture was affected by the presence of contralateral disease, despite being carefully clinically examined and found to be Normal. As is outlined in the methods section, primers were used to identify various bacterial groups using RT-PCR. We adapted and partially validated (using dilutions of a pure culture of *M.bovis*, but not *M.bovoculi*) the use of primers from Zheng et al. [63] for identification of *Moraxella bovis*. Additional validation would be required to assess specificity of the *Moraxella* primers which were utilized in the present study. Despite concerted efforts, a suitable standard to validate the *Weeksellaceae* primers (identified using predictive local alignment) was not identified. As such, the values from this component of the RT-PCR panel cannot be considered to be specific for *Weeksellaceae*. Finally, IBK was diagnosed based on the presence of compatible clinical signs, rather than the presence of a single organism (e.g., *Moraxella bovis*). This element of study design was deliberate, based on previously discussed evidence that *Moraxella bovis* is not present in many suspected cases of IBK [1, 14, 16, 40].

Sampling bias also represents a possible limitation of the present study. Affected herds for sampling and inclusion were identified by specifically seeking animals with IBK. Therefore, all farms which were visited during the sample collection phase were known to have IBK affected cattle and as such, comparison statistics reported herein involving demographics and disease status are likely to be impacted by sampling bias. In addition, Normal eyes from IBK unaffected cattle (cattle with two Normal eyes) were sampled at each location, and the number of Normal cattle sampled at each location was not consistent due to animal availability. Sampling bias may therefore explain certain statistically significant findings, such as the higher number of IBK-affected eyes in male cattle compared to Normal eyes in male cattle. Sampling bias also likely contributed to the number and types of breeds included in the study: a large number of Angus cattle affected by IBK were included when compared to breeds such as Hereford cattle. Angus cattle have been studied for genetic predisposition to IBK development [64], though Hereford cattle are a breed considered to have high susceptibility and increased predisposition, theorized to be related to periocular pigmentation or heredity [6, 65, 66]. A large variation in sample sizes of male cattle and female cattle sample may be related both to sampling biases described above as well as to an unbalanced ratio of male and female cattle across the United States [67]. Finally, a large variation in sample size with regard to age may be related both to sampling biases described above as well as to a previously documented predisposition for this disease to affect young calves [5].

## Conclusions

This study provides further evidence that the bovine bacterial OSM is altered in the context of IBK, indicating the involvement of a variety of bacteria in addition to *Moraxella bovis*, including *Moraxella bovoculi* and *R. nasimurium*, among others. *Actinobacteriota* relative abundance is altered in IBK, providing opportunities for novel therapeutic interventions. While RT-PCR modeling provided limited further support for the involvement of *Moraxella bovis* in IBK, this was not overtly reflected in culture or RA results. RT-PCR modeling demonstrates potential as a cost-effective method to reliably confirm IBK.

## Methods

### Subject selection and examination

**Fig. 7:**
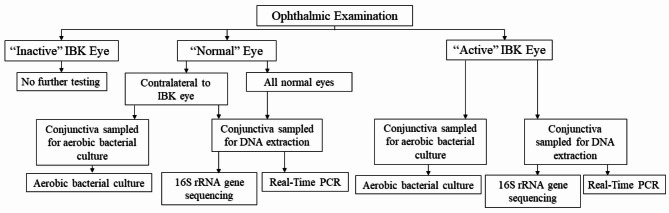
Flow chart of the experimental design for evaluation of the bovine bacterial OSM, including initial ophthalmic examination, diagnosis of active IBK, inactive IBK, or normal eyes, and the specific subsequent samples collected for further analysis

The study was approved by the Louisiana State University (LSU) Institutional Animal Care and Use Committee (Animal Use Protocol 19–092). A flow chart for visualization of the experimental process may be found in Fig. 7. Outbreaks of IBK were identified by telephone calls and emails to veterinary schools, veterinary practices, and producer organizations within the United States. Herd examinations and sample collection were performed between May and October 2021. Consent of the herd owner or equivalent representative was obtained prior to performing conjunctival sampling of the eyes of affected and normal cattle. All animals had received no treatment in the 7 days prior to examination and sample collection. Where possible, visits were made in conjunction with a visit by the local responsible veterinarian who treated the animals immediately following conjunctival sample collection. Calves and adult cattle of both dairy and beef purpose were included in the study, with breed and age recorded as reported by farm personnel. Age was divided into three categories of < 1 year old, 1–5 years old, and over 6 years old. Cattle for dairy production were housed in open-air barns and were handled frequently, while cattle for beef production were on pasture and handled relatively infrequently.

### Sample collection

Ophthalmic examination was performed by a trained veterinary observer. Adult cattle were held in a chute with a head catch for examination, while younger calves were physically restrained by farm staff. The eyes were categorically diagnosed as ‘Normal’ or ‘IBK’. Only eyes with evidence of active IBK were included in the IBK group. An eye with two or more of the following clinical signs in the context of a herd of two or more cattle with similar signs was considered to have active IBK disease: blepharospasm, epiphora or ocular discharge, ulcerative keratitis, chemosis, conjunctival hyperemia, corneal vascularization, corneal edema, corneal infiltrate, and corneal perforation with or without iridial prolapse. Eyes with evidence of corneal scarring without active inflammation (inactive IBK) were not included in the study population. Only eyes diagnosed as IBK or Normal were included, and therefore both eyes of some cattle were not included due to the presence of inactive IBK. Following sample collection, eyes were evaluated as individual entities and were not kept paired for analysis. Animals with clinical signs indicative of a possibly unrelated ocular disease process (e.g. exophthalmos) were excluded from the study population.

If at least one eye of an animal was determined to be Normal, the lower conjunctival fornix of the normal eye(s) was sampled vigorously with two DNA buccal swabs (Isohelix Swab Pack, MidSci, St Louis, MO) simultaneously. The swabs were placed in a 15 mL centrifuge tube (VWR, Radnor, PA) prior to being placed on ice for storage for later DNA extraction. If at least one eye of an animal was determined to be actively affected by IBK, the lower conjunctival fornix of the IBK eye was sampled vigorously with DNA buccal swabs (Isohelix Swab Pack, MidSCi, St Louis, MO) followed by a bacterial culture swab (Copan Diagnostic ESwab, Copan Diagnostics, Murrieta, CA) with each swab type then placed in its respective tube. If the contralateral eye of an IBK eye was also diagnosed as active IBK or Normal, both eyes were sampled for bacterial culture. If the contralateral eye was diagnosed as inactive IBK, only the active IBK eye was sampled for bacterial culture. If both eyes of an animal were diagnosed as Normal, neither eye was sampled for bacterial culture. The samples were then stored on ice for later DNA extraction and to be sent off for culture, respectively. Non-sterile gloves (VWR, Radnor, PA) were worn and changed between each animal during sample collection. An environmental control for use in relative abundance analysis was created at each sampling location by exposing two DNA buccal swabs to the air for approximately five seconds with immediate storage in a 15 mL tube on ice for later DNA extraction. All samples for DNA extraction were shipped to LSU School of Veterinary Medicine directly from the farm. Samples for bacterial culture were shipped directly to the Texas Veterinary Medical Diagnostic Laboratory (TVMDL) for processing.

### Bacterial culture

Culture swabs were shipped on ice directly from sampling locations to the TVMDL for aerobic culture. For isolation of bacteria, conjunctival swab samples (n = 387) were streaked onto two 5% sheep blood agar (Hardy Diagnostics, USA) plates and incubated aerobically with 10% CO_2_ at 37 °C for 48 h. All the culture plates were read at 24 and 48 h for isolation of bacteria. Initial identification of different bacteria were based on colony morphologies on plates, and different biochemical test results including oxidase, catalase, indole, carbohydrate fermentation and gram staining. Gram stain and oxidase (BD Diagnostics, USA) tests were performed on all the *Moraxella* spp. suspected isolates producing greyish-white and hemolytic colonies. *Moraxella* spp. are gram negative cocci and oxidase positive. Finally, identification of different bacteria up to genus or species level were based on matrix-assisted laser desorption-ionization time of flight mass spectrometry (MALDI-TOF MS) (Bruker Daltonics, Germany) score results. A score of 2.3 to 3.0 was considered a highly probable species identification, and a score of 2.0 to 2.299 was considered a secure genus identification and probable species identification. Final identification of all bacterial isolates was based on the agreement between biochemical and MALDI-TOF MS results. When mixed bacterial colonies with no predominant colony types were present on bacterial culture plates, these were reported as ‘mixed bacterial growth’. For the samples when *Moraxella* spp. were not isolated, those were reported as “negative culture *Moraxella* spp.”

### DNA extraction

DNA Extraction from each swab used for 16s rRNA gene sequencing and RT-PCR analysis was performed using the DNeasy PowerSoil Pro Kit (QIAGEN GmbH, Hilden, Germany) according to the manufacturer’s instructions. DNA was extracted from conjunctival swabs, environmental control samples, and from extraction control samples created by replicating the protocol in the absence of swabs. A filtered laminar flow cabinet (The Clone Zone, USA/Scientific, Inc., Ocala, Florida, USA) was used to perform the extractions. Eluted DNA sample concentrations were calculated (NanoDrop One Microvolume UV-Vis Spectrophotometer, ThermoFisher Scientific, Waltham, MA), and samples were stored at -80 °C.

### 16 S rRNA gene sequencing and analysis

Sequencing was performed by the LSU School of Medicine Microbial Genomics Resource Group. The AccuPrime Taq high fidelity DNA polymerase system (Invitrogen, Carlsbad, CA) (Table 6) was used to perform two steps of amplification for sequencing library preparation. Amplicon library preparation included processing negative controls (DNA extraction, environment, and PCR amplification) and a positive control (Microbial mock community HM-276D, BEI Resources, Manassas, VA) (Supplemental Table 1). Twenty nanograms of genomic DNA and gene-specific primers (F: GTGCCAGCMGCCGCGGTAA, R: GGACTACHVGGGTWTCTAAT) with Illumina adaptors were used to amplify the hypervariable V4 region. PCR included steps listed in Table 7. AMPure XP beads with beads added as 0.85x the PCR volume were used to purify PCR products (targeting approximately 390 bp DNA). Using the same PCR conditions and primers with different molecular barcodes, 4 μL of purified amplicon DNA from the previous step was amplified for 8 cycles. AMPure XP (Beckman Coulter, Indianapolis, IN) beads were used to purify the indexed amplicon libraries, which were then quantified using Quant-iT PicoGreen (Invitrogen), normalized, and pooled. KAPA Library Quantification Kit (Kapa Biosystems, Cape Town, South Africa) was used to quantify the pooled library, followed by dilution and denaturation as per Illumina guidelines. As a quality control and to increase diversity of the 16 S rRNA amplicon library, ten per cent Illumina PhiX was added to the sequencing library. A 2 × 250 bp V2 sequencing kit was used to perform paired-end sequencing using Illumina MiSeq (Illumina, San Diego, CA). Quality analysis was performed through transfer of the sequencing reads to Illumina’s BaseSpace. Further bioinformatics analysis was performed with the generated raw FASTQ files.

**Table 6:** PCR Master Mix components utilized in PCR prior to 16 S rRNA gene sequencing

AccuPrime ™ *Taq* DNA Polymerase System
10X AccuPrime™ PCR Buffer II (500 μL) containing:
• 200 mM Tris-HCl (pH 8.4) • 500 mM KCl • 15 mM MgCl_2_ • 2 mM dGTP • 2 mM dATP • 2 mM dTTP • 2 mM dCTP • Thermostable AccuPrime™ protein • 10% glycerol • 50 mM Magnesium Chloride (500 μl)

**Table 7:** Cycling process utilized in PCR prior to 16 S rRNA gene sequencing. The total reaction volume used was 20μL with 20ng of sample DNA

Run Stage	Temperature	Time
Step 1	95℃	3 min
Step 2: (25 cycles)	95℃	30 s
Step 3	55℃	30 s
Step 4	72℃	30 s
Step 5	72℃	5 min
Step 6: Hold	4℃	

Sequencing reads from FASTQ files were imported into R version [68] 4.2.0. Reads were then processed with DADA2 [69] version 1.22.0. Read quality profiles were examined to select appropriate trimming and filtering parameters and were set to trim 20 bp (left) of each read and to truncate reads to 240 bp (both forward and reverse) to remove low quality tails. The standard DADA2 workflow, including error learning and sample inference for forward and reverse reads followed by merging of sequence variants, was utilized. The ‘removeBimeraDenovo’ process was used to remove chimeric sequence variants, and sequence variants outside of the expected amplicon size range of 249 to 256 bp were removed as well. The remaining sequence variants were placed into a sequence table with read counts ranging from 816 to 143,459. The SILVA database v138 [70] was used to classify taxonomy, and a Phyloseq [71] object was constructed using imported mapping information. Phyloseq [71] version 1.38.0 was used to perform downstream analysis. Decontam [72] version 1.14.0 was used to identify and remove suspected contaminant ASV with the prevalence method (default parameters). Remaining ASVs with a mean relative abundance of less than 10^− 4^ across all samples were filtered with an abundance filter.

### Real-time PCR

RT-PCR for selected bacterial familes and species (Table 8) was performed using remaining eluted DNA and PerfeCTa SYBR Green FastMix, ROX (VWR, Radnor, PA) (Table 9). Bacterial target primer pairs were chosen based on previous clinical reports and exploratory 16S rRNA gene sequencing relative abundance analyses obtained from cattle with IBK [9, 42, 63]. Eluted DNA concentrations from eyes of the same animal were standardized through dilution with molecular grade water (VWR, Radnor, PA) with an overall range of 0.3 to 34.3 ng/μl. DNA samples were individually combined with nine different primer pairs and analyzed in triplicate. The RT-PCR panel performed included the following primer (IDT, Coralville, IA) targets for each sample: bovine GAPDH [73], *Moraxella bovis* [63], *Moraxella bovoculi* [63], *Mycoplasma* [74], *Pasteurellaceae* [75], *Prevotellaceae* [76], *Staphylococcus* [77], *Weeksellaceae*, and ‘universal bacteria’ [35]. The universal bacteria primer was used for data normalization, while the bovine GAPDH primer was utilized as a reference for host DNA in proportion to the total bacterial DNA extracted. *Escherichia coli* standard (10 ng/μL, Sigma-Aldrich, Saint Louis, MO) and molecular grade water (VWR, Radnor, PA) were used as positive and negative controls, respectively. To allow for standardization between runs and relative quantification of non-E. *coli* bacterial groups, the *E. coli* standard was plated in triplicate with both ‘universal bacteria’ primers and *E. coli* primers [78]. The RT-PCR run consisted of steps listed in Table 10. Using an *E. coli* standard on the same RT-PCR plate, the RT-PCR data was expressed as the log amount of DNA in atto-gram (10^-18^ g, ag) for each primer pair per 10 ng of isolated total DNA [79]. Within each sample, the C_T_ values of each primer set were normalized by universal bacteria C_T_ values for further analyses.

**Table 8:** A novel RT-PCR primer panel for detection of IBK. Normalization was performed using the ‘universal bacteria’ and *Escherichia coli* primer sets

Target	Forward primer	Reverse primer	Reference
Bovine GAPDH	CCTGGAGAAACCTGCCAAGT	GCCAAATTCATTGTCGTACCA	[73]
*Moraxella bovis*	GGTGACGACCGCTTGTTT	ATCATCGCCTTCATCTCCAG	[63]
*Moraxella bovoculi*	GGTGATATTTATCATGAAGTTGTGAAA	TYTCAATTCATAATCACGATACTCAAG	[63]
*Mycoplasma*	TGCACCATCTGTCACTCTGTTAACCTC	ACTCCTACGGGAGGCAGCAGTA	[74]
*Staphylococcus*	GGCCGTGTTGAACGTGGTCAAATCA	TIACCATTTCAGTACCTTCTGGTAA	[77]
*Pasteurellaceae*	CATAAGATGAGCCCAAG	GTCAGTACATTCCCAAGG	[75]
*Prevotellaceae*	GGTTCTGAGAGGAAGGTCCCC	TCCTGCACGCTACTTGGCTG	[76]
*Weeksellaceae*	ATCCAGCCATCCCGCGT	CTGCTGGCACGGAGTTAGC	None; novel
Universal Bacteria	CCTACGGGAGGCAGCAGT	ATTACCGCGGCTGCTGG	[35]
*Escherichia coli*	CCGATACGCTGCCAATCAGT	ACGCAGACCGTAGGCCAGAT	[78]

**Table 9:** PCR FastMix components utilized in RT-PCR

PerfecCTa SYBR® Green FastMix
• Reaction buffer with optimized concentrations of molecular-grade MgCl_2_, dATP, dCTP, dGTP, and dTTP
• AccuStart II Taq DNA Polymerase
• SYBR Green I dye
• Proprietary enzyme stabilizers and performance-enhancing additives

**Table 10:** Cycling methods for RT-PCR, including run stage with associated temperature and duration

Run Stage	Temperature	Time
Hold	50℃	2 min
	95℃	10 min
PCR (40 cycles)	95℃	15 s
	60℃	10 min
Continuous melt curve	95℃	15 s
	60℃	15 s
Dissociation	95℃	15 s

### Classification analysis via random forest algorithm

The C_T_ values of eight primer sets (Bovine GAPDH included) were normalized by the universal bacteria primer C_T_ values generated for the same sample. The data was further analyzed using a random forest algorithm [80] to categorize Normal and IBK samples using commercial software (JMP Pro 16.2.0). The number of ‘trees per forest’ was set to 100 with the early stopping option allowed. The number of predictors sampled at each split was 6 with minimum and maximum splits per tree set at 10 and 200. Parameters (sensitivity and specificity from the validation sets and the relative deviance (G^2^) from the training set) were reported with 95% confidence limit generated via 2500 runs. Approximately 80% (n = 459–498) and 20% (n = 98–137) of the total data were used for building the training model, and validation set, respectively. Sensitivity and specificity values were calculated from each run for the training and validation sets separately.

### Statistical analysis

Commercial software (JMP Pro 16.2.0 [81] and R Statistical Software v4.1.3 [68]) was used to perform all statistical analyses. Chi-squared test was used to check associations between culture results, eye sampled (right or left), geographic location, and disease status. One way ANOVA and student’s t tests were used to evaluate abundance against disease state, geographic location (state and farm), sex, age, and breed. Logarithmic transformation was performed for data that did not meet the normality criteria. Normality of residuals from the parametric models were accessed and confirmed by examining standardized residual and quantile plots. Data are presented as mean ± SD. Kruskal-Wallis tests or Mann-Whitney tests against disease status, geographic location, breed, and sex were used to analyze alpha-diversity from 16 S rRNA gene sequencing, and Log DNA concentration from RT-PCR. Beta-diversity indices (standard weighted unifrac analysis, unweighted unifrac analysis, and Bray-Curtis analysis) were evaluated via permutational multivariate analysis of variance (PERMANOVA) using vegan R package 2.6.2 [82]. Statistical significance was set at *p* < 0.05.

### Electronic supplementary material

Below is the link to the electronic supplementary material.

**Supplementary Material 1: Supplemental Table 1**: Number of reads before and after filtering per sample. Input = original number of sequences, prior to filtering, Filtered = number reads following initial quality filtering, DenoiseF = number of reads remaining after denoising forward, DenoiseR = number of reads remaining after denoising reverse, Merged = number of reads remaining after merging forward and reverse reads, Nonchim = number of merged reads remaining after chimera removal

**Supplementary Material 2: Supplemental Table 2**: Relative abundance data by state. N = number of eyes

**Supplementary Material 3: Supplemental Table 3**: Relative abundance data by farm. N = number of eyes

**Supplementary Material 4: Supplemental Table 4**: Relative abundance data by individual breed. N = number of eyes

**Supplementary Material 5: Supplemental Table 5**: Relative abundance data by breed purpose. N = number of eyes

**Supplementary Material 6: Supplemental Table 6**: Beta diversity subgroup analysis

## Data Availability

The datasets generated and/or analyzed during the current study are available in the National Center for Biotechnology Information Sequence Read Archive repository, https://www.ncbi.nlm.nih.gov/sra/PRJNA987343, https://www.ncbi.nlm.nih.gov/sra/PRJNA987346.

## List of abbreviations

16S rRNA: 16 S ribosomal ribonucleic acid
Ag: Atto-gram, 10^− 18^ g
DNA: Deoxyribonucleic acid
IBK: Infectious bovine keratoconjunctivitis
MALDI-TOF MS: Matrix-assisted laser desorption-ionization time of flight mass spectrometry
OSM: Ocular surface microbiome
RT-PCR: Real-time polymerase chain reaction
TVMDL: Texas Veterinary Medical Diagnostic Laboratory
